# Acupuncture for Treatment of Autism Spectrum Disorders

**DOI:** 10.1155/2012/679845

**Published:** 2011-12-06

**Authors:** Xue Ming, Xiang Chen, Xiao T. Wang, Zhen Zhang, Victor Kang, Barbie Zimmerman-Bier

**Affiliations:** ^1^Department of Neurosciences and Neurology, UMDNJ-New Jersey Medical School, 90 Bergen Street, DOC 8100, Newark, NJ 07103, USA; ^2^Department of Rehabilitation, Yu-Yin Children's Hospital, WenZhou Medical College, WenZhou 325035, China; ^3^Office of Research and Sponsored Summer Student Program, UMDNJ-New Jersey Medical School, Newark, NJ 07103, USA; ^4^Marlboro High School, Marlboro, NJ 07746, USA; ^5^Department of Pediatrics, St. Peter's Hospital, New Brunswick, NJ 08901, USA

## Abstract

*Background*. There has been lack of reviews of evidence on efficacy, methodology, and/or safety of acupuncture in autism spectrum disorders. This paper examines the emerging evidence of the effects of acupuncture in the treatment of autistic children. *Method*. A literature review was completed via Medline and three Chinese search engines. A total of 31 studies were evaluated for acupuncture methodology, study design, treatment effects, and tolerability. *Results*. The acupoints used, the duration of needling, the frequency of treatment, the choice of stimulation, and the course of the treatment were highly variable amongst the studies. Behavioral and/or developmental improvements were reported in all acupuncture treatment studies. All studies reported general tolerability. Weakness of experimental designs was discussed. *Conclusions*. Vigorously controlled double-blinded clinical trials are needed to evaluate the efficacy and safety of acupuncture in children with autism spectrum disorders.

## 1. Introduction

Acupuncture has been used in the treatment and prevention of illnesses for over two thousand years. The basic theory of acupuncture starts with the meridian system and flow of energy, or *qi*. There are twelve regular and eight supplemental meridians that run either longitudinally along the body surface or from the interior to the exterior of the body to the surface of the body. There are many smaller collaterals off each meridian. The meridians cover all parts of the body and are divided into *Yin* and *Yang* meridians that are distributed in symmetry on the body surface. Meridians connect Zang-Fu organs to each other and to the surface of the body. Zang-Fu organs, such as the liver, heart, spleen, and kidneys, are not the anatomical corresponding organs classified in western medicine. The brain is connected with all of the Zang-Fu organs. The balance of *Yin* and *Yang* in Zang-Fu organs connects health or disease states with the meridian systems. Acupuncture can be used to regulate the Zang-Fu organs' functions via the flow of *qi* and *xue* (blood) through the meridian system. The flow of *qi* and *xue* is essential for good health. Overcoming obstructions of *qi* and/or *xue* is a goal of acupuncture [1]. 

 In order to make a treatment plan based on acupuncture, a doctor will need to make a diagnosis based on four components: inspection, auscultation, inquiry, and palpation. For example, the doctor inquires about symptoms, then looks at the tongue, feels the pulse, and looks at the hair and skin color, as well as the general condition. The doctor then determines which Zang-Fu organ(s) is affected. The doctor chooses the acupoints which regulate the affected Zang-Fu organ(s). These could include acupoints close to the Zang-Fu organs or distant acupoints that are connected via meridians. Once acupoints are chosen, the doctor determines the angle, depth, and duration of needle placement and if electrical or manual stimulation should be applied to the needle to intensify the treatment. Other treatment decisions include the frequency and length of acupuncture administration. Treatment plans can be modified depending on individual patient responses. Those with chronic illnesses may require repeated courses of acupuncture treatment combined with moxibustion and Chinese massages in order to achieve maximal benefit. 

 Western medicine's study of acupuncture, while still in its infancy, has led to greater understanding and acceptance of it. For example, it is believed that the pathways of meridians bear a close relationship with the peripheral nerve distribution [2]. The spread of the needling sensation from an acupoint or the effect of analgesia is blocked when there is a sensory nerve injury around the acupoint [1, 3]. Stimulation of median nerves underlying P5-P6 acupoints increases the activity of the arcuate nucleus and provokes the activity in the ventrolateral periaqueductal grey matter (which ultimately regulates premotor sympathetic outflow) leading to lowering blood pressure [4–7]. It is believed that an endorphin-like substance is released during the course of acupuncture analgesia, and the effect of this substance is blocked by the opiate antagonist naloxone [8]. Pharmacological studies revealed that the analgesic effects associated with electrical acupuncture are mediated by *mu* and *delta* opioid receptors [9]. Changes in the hypothalamus-pituitary-adrenal axis were associated with anti-inflammatory effects of electrical acupuncture [10]. Functional MRI studies demonstrated multiple cortical, subcortical/limbic, and brainstem activation during acupuncture in humans [11–14]. Further research is needed to understand how the autonomic nervous system or central nervous system is regulated by acupuncture. 

Acupuncture has shown treatment success in a variety of illnesses. Acupuncture is frequently used in China to treat chronic joint inflammation, local pain syndrome, headache, insomnia, facial palsy, neuralgia, stroke, language disorders, cardiovascular diseases, asthma, gastrointestinal dysfunction, incontinence, impotence, gynecological diseases, and so forth. In the United States, the National Institute of Health published a consensus statement on the use of acupuncture as an adjunct treatment or alternative in a comprehensive management program for certain disorders [8]. In November 2007, the Society for Acupuncture Research hosted a follow-up international conference to evaluate the progress of acupuncture research. Based on published literature, the efficacy of acupuncture is well defined in treatment of chronic pain, inflammatory disorders, and gynecological disorders and is less defined in treating neurological or mental disorders [10]. 

 In recent years, there has been an increase in the development of rehabilitation centers in China. Acupuncture has been used in these centers as part of treatment programs for children with developmental disorders including speech and language delay, cerebral palsy, mental retardation, ADHD, and autism. This author (XM) attended The Fourth National Conference on Child Rehabilitation and Cerebral Palsy held in Wen Zhou, China in June 2010. At the conference, there were reports of varying degrees of improvement in motor and/or language function in children with cerebral palsy when acupuncture was added to traditional physical, occupational and speech therapies. No single report of acupuncture treatment in autism spectrum disorders was presented at the conference despite many reports of behavioral or biomedical interventions for autism. Prompted by the effects of acupuncture in neurodevelopmental disorders and the paucity of presentations on acupuncture in autism, a review of scientific literatures began with collaboration of authors from the USA and China. The objective of this review is to evaluate clinical studies for efficacy and safety of acupuncture in the treatment of behavioral and developmental disorders of autism spectrum disorders.

## 2. Method

A descriptive review of acupuncture in the treatment of autism spectrum disorders was conducted by literature search. Keywords “acupuncture,” “acupressure,” “acupoint,” “electroacupuncture,” or “meridian” combined with “autism” and “autism spectrum disorders” were conducted via Medline and the Chinese search engines, Chinese Network Knowledge Infrastructure (CNKI, 1979–2011), Chinese Scientific Journal Database VIP (1989–2011), and Wan Fang Database (1985–2011). The inclusion criteria for selection of clinical studies for this review were (1) human clinical studies in children with autism spectrum disorders of ages 1 to 18 years, (2) description of acupuncture methodology (i.e., acupoints used), and (3) efficacy data presented. Forty articles met the criteria, 35 of the articles in Chinese (21 of them with English abstracts) and 5 articles in English. Duplicate publications resulting from a study were not counted as original studies. All of the Chinese articles were interpreted by X. Ming and Z. Zhang, both authors are fluent in Chinese. All articles (case studies, review articles, and commentary and original research studies) were reviewed for acupuncture methodology, safety, efficacy, and application. The quality of the clinical trials was further analyzed through assignment of a Jadad score [15]. The results of the clinical trials are descriptively summarized. In addition, studies with over 50 subjects or Jadad score 2 or more were summarized in a table format. 

## 3. Results

Of the 31 studies reviewed, there were 27 original research studies: 24 [16–42] in Chinese and three [43–45] studies in English (one performed in Egypt, the other two in Hong Kong). Four articles originated from one study but analyzed different clinical characteristics and/or outcome measures [16, 19, 23, 24] and were regarded as one original study. Four case studies [46–49] were reviewed for methodology, efficacy, and/or safety. In addition, there were four review articles [50–53] and two commentary articles on application of acupuncture in autism [54, 55]. 

The 27 original clinical trials were rated by the Jadad scale [15]. One study [33] was rated as Jadad score of 5, two study [22, 45] received a score of 3, four studies [25, 30, 41, 44] rated 2, six studies [16, 19, 21, 23, 24, 31, 32, 37, 39] attained the score of 1, and fourteen studies [17, 18, 20, 26–29, 33, 34, 36, 40, 42, 46, 48] scored 0. A summary of the subject numbers, whether control groups were used, randomization, blinded assessment, description of drop-outs, and Jadad scores are shown in Table 1. 

### 3.1. Acupoints and Acupuncture Method Used in the Studies

The acupoints and acupuncture method used in the studies are summarized in Table 2.

Of the 31 clinical studies (original research and case studies) [15–49], 25 used predominantly scalp acupoints along with supplementary body acupoints [16–33, 36, 39–42, 47, 48], two studies used only acupoints on the body surface [34, 35], three studies [34, 38, 49] used similar amount of scalp and body surface acupoints, and two studies used tongue acupoints [39, 44]. 

Ten studies [16–25, 27, 29–32] used Jin's three-needle system as the primary acupoints for treatment of autism spectrum disorders. Jin's three-needle system includes tongue acupoints. These studies also used two to three additional supplementary acupoints based on individual subject's symptom reading {Traditional Chinese Medicine (TCM) syndrome}. The Jin's three-needle system acupoints used were *sishenzhen (four conscious needles), niesanzhen (three temporal needles), naosanzhen (three brain needles), touzhizhen (head wisdom needle), shesanzhen (three tongue needles), shousanzhen (three hand needles), shouzhizhen (hand wisdom needle), zusanzhen (three foot needles), *and* zuzhizhen (foot wisdom needle). *Studies that did not use Jin's three-needle system [26, 28, 30, 31, 33, 36–47] showed no consensus on the selection of acupoints (especially on body acupoints). Among these studies, *sishenzhen*, *Baihui, naomeng, yintang, *and* shenting were frequently used *[26, 28, 30, 31, 33, 36–38, 43, 45–47]. Specific tongue acupoints developed by the acupuncturist Dr. Sun were used in their study [44], and tongue acupoints for brain, heart, liver, and kidneys were used in the other study [39]. 

The depth and angle of needle puncture, the duration of needling, and the frequency and duration of treatment course were highly variable among studies. The depths and angles of the needles varied with the specific acupoints used. The depth of needling ranged from 0.2 to 1 *cun (Chinese inch). *The depth of the needling depended on the angle of penetration, that is, the depth was deeper when the needle angle was flatter, and shallower when the needle was perpendicular to the skin. The angles were usually 0 degrees, 45 degrees, 90 degrees, or a specific angle from the acupoint towards a landmark anatomic site [16, 19, 23, 24]. 

The duration of needling also varied amongst studies and by acupoints within the same study. Some used immediate withdrawal after puncture [16, 19, 23], and others leaving the needles in for less than 15 seconds [44], 10 minutes [18, 21], 15 minutes [46], 25 minutes [30], 30 minutes [21, 25, 30, 32, 48], 45 minutes [18, 19, 23, 24, 33], 60 minutes [47], or even 2–4 hours [41, 42]. Some studies stated a range of duration such as 30–60 minutes [17, 33, 38], or a varied duration depending on the acupoints [19, 23]. Some studies did not specify the duration of needling [20, 28, 34, 36]. Two studies used only acupoints massage [37, 49]. 

 The frequency and duration of the treatment were also highly variable. The frequency of treatment per week varied from one to six sessions per week [16, 18, 19, 23, 24, 33, 41], five sessions [25, 30, 31, 39, 44], four sessions [29], three sessions [28, 42], two sessions [22, 45, 47], to weekly [40]. The length of treatment was also highly variable and ran from four weeks to nine months. Three studies did not specify the duration of entire treatment period [21, 38, 47]. Some studies used four cycles of treatment, 12 weeks per cycle with four weeks rest intervals. The acupoint massage studies used one minute to each acupoint daily for four months [37], or an undefined length of daily massage over 12 days [47]. 

In addition to needle penetration, five studies used electrical stimulation [28, 29, 31, 48, 51], nine used manual stimulation [16, 17, 19, 20, 23, 24, 33, 45, 47], and two used injection of vitamins into acupoints [36, 51].

### 3.2. Subjects

The age of subjects in the studies reviewed ranged from as young as 1.5 years to as old as 18 years. Most of the studies were performed in children under 12 years old. The average age among most of the studies was six years. There was a male predominance in all the studies reviewed, which is consistent with the recognized male predominance of autism (4 : 1 male to female) [56]. The diagnosis of autism was based on DSM IV or ICD-10 criteria. One study used an additional criterion, autism diagnostic interview-revised (ADI-R) [44], and another one used both ADI-R and autism diagnostic observation schedule (ADOS-G) [43]. None of the studies reviewed had specified the autism spectrum diagnosis included. The number of the participating subjects ranged from 11 to 202 among the original research studies and one to two subjects in case reports. 

### 3.3. Study Designs and Measurements

#### 3.3.1. Study Design

There were 17 studies that used a comparison group [16–26, 30–32, 37, 39, 41, 43–45]. The comparison groups were autistic children receiving behavioral therapy, Chinese herbal medicine, Chinese massage, or a combination of behavioral therapy and herbal medicine. Some studies used supplementary TCM along with the acupuncture in the same subjects concurrently or sequentially. One study used a combination of acupuncture, Chinese herbal medicine, Chinese massage, and behavioral therapy [33], rendering the interpretation of results difficult. A better study design reviewed compared the treatment effect in three independent groups of acupuncture, behavioral therapy, or Chinese massage therapy [26]. Another two studies were conducted by the injection of vitamins into the scalp acupoints [35, 36]. Some studies used acupuncture and behavioral therapy simultaneously within the same subjects [21, 31]. One study used massage at acupoints without actual needling [37]. Another study used both acupuncture and acupoints massage [49]. 

 There were one double-blinded studies [43] and three single-blinded study [41, 44, 45]. Of the double-blinded studies, both the parents and the outcome assessors were blinded (sham controls were used). Of the single-blinded studies, the assessors were blinded. 

#### 3.3.2. Outcome Measures

The outcome measures varied amongst studies and were largely behavioral scales but also included measures of language and development. These behavioral scales rate behaviors such as social interaction and awareness, language and communication, fine motor coordination, self-care, cognition, and aberrant behaviors. The behavioral scales used included childhood autism rating scales (CARS), autism behavioral checklist, clinical global impression-improvement scale, clancy autism behavior scale, and aberrant behavioral checklist (ABC). The language, cognitive, and motor developmental scales included the psychoeducational profile (C-PEP) Chinese Version, Peabody picture and vocabulary test (PPVT), sign signification relations, the intelligent quotient (IQ) test, the autism development checklist (ADC), an arabic language test, the Ritvo-Freeman real life scale, WeeFIM, the WPPSI, the WISC-R, and the Gesell development scale. Three case reports used clinical anecdotal impression to report the results [46, 47, 49]. One study used improvement in evoked potential P3 latency/amplitude [25], while another study used improvement in single-photon emission computed tomography (SPECT) brain blood flow [34], to measure the treatment effects of acupuncture.

All 27 original research studies used a statistical method for data analysis. 

### 3.4. Treatment Effects

Statistical improvement in behavior and/or development in children with autism treated with acupuncture were reported in all the studies reviewed. In the 17 studies [16–26, 30–32, 37–39, 41] that used a comparison group, acupuncture-treated groups were reported to have a superior improvement over behavioral therapy-treated groups, Chinese herbal treatment groups, music treatment, or Chinese massage treatment groups (*P* < 0.05, *P* < 0.01). When there were a combination of treatments in the same subjects, acupuncture plus the comparison modality of treatment was claimed to be superior than the comparison treatment alone [21, 30, 31, 41, 45]. Ma et al. [21] used three groups in their study design, acupuncture, behavioral therapy, and combined acupuncture and behavioral therapy. They reported that the combination group had a greater improvement than either acupuncture or behavioral therapy alone (*P* < 0.01). In the studies that used sham controls, significantly greater improvements of the outcome scores before and after treatment in acupuncture-treated groups over control groups were reported (*P* < 0.0005, *P* < 0.01) [43, 44]. All of the ten studies [27–29, 33–36, 38, 40, 42] without a comparison group reported improvement after acupuncture treatment, among them, three studies reported statistically significant improvement [28, 29, 34] (*P* < 0.05, *P* < 0.01). Likewise, the four case studies reported improvement after acupuncture [46–49].

There were no specific domains of development that were reported to be uniformly improved among the studies. The reported improvements among the studies include better language communication function and/or social interaction, reduction of repetitive behaviors, improvements in fine motor, self-care, and/or cognitive function. In the studies that reported the proportion of children with autism improved (all degrees of improvement), the rates of improvement were over 80% in the acupuncture-treated groups versus over 50% in the comparison groups [16–26, 30–32, 36, 45]. The improvements in the treatment groups were reported to be statistically greater than the comparison groups in all these studies. However, some studies [16–18, 20–24, 26–28, 30–32, 37, 45] reported a degree of improvement such as “dramatic improvement,” “improvement,” and “no improvement,” and the results were quite variable amongst studies. The degree of improvement was largely based on an assigned degree of improvement in scores. Such an assigned degree of improvement was not uniform across studies. Of the studies that reported some degree of improvement [16, 17, 19, 23, 24, 29, 31, 33], subjects with “dramatic improvement” ranged from 33 to 97%; those with “improvement” were reported from 9 to 63%; from 0 to 21% subjects showed “no improvement.” 

A summary of studies with 50 or more subjects or Jadad scores of 2 or more is presented in Table 3 in the form of the number of subjects, type of study, length of study, outcome measures, and the results (Table 3). 

Only one of the 27 studies reported a clinical follow-up. Liu et al. [28] reported a 12-month followup of the subjects. Of the 30 subjects that showed improvement, 14 subjects continued to improve and nine of the 14 subjects integrated to mainstream class. Ten subjects did not show further improvement, while six subjects showed regression. This study did not use a comparison group; the outcome measures used were CARS and a Chinese social function evaluation instrument evaluation.

### 3.5. Tolerability

A few studies that reported tolerability to acupuncture stated that the participating autistic children tolerated the procedure and had no significant side effects [28, 35, 43, 44, 48]. Local brief bleeding was associated with needling [43, 48]. No infection was reported. However, some studies [16–19, 23, 24, 28, 31, 48] stated that the subjects were preselected based on predictions of being tolerable to the procedure; therefore, the studies were not random to any child with autism, and caution should be exercised in the interpretation of the tolerability. Yuan et al. [15, 19, 23, 24] stated that the subjects that had significant side effects or those not willing to continue treatment were excluded from the study. However, there was no description of what the significant side effects were or how many of these children were excluded. Wong et al. [43] reported more than 70% of the subjects adapted to acupuncture easily, while 8% had poor compliance. Anecdotal experience of many acupuncturists showed nil significant side effects, better tolerability in autistic children whose parents had a strong desire for the treatment, and improved tolerance after a few sessions of treatment (personal communication). None of the studies reported use of restraints or sedation for the procedures in the autistic children.

## 4. Discussion

All of the reviewed articles invariably reported improvement in autistic children who received a form of acupuncture with or without concurrent behavioral intervention. All the clinical trials reported superior effects of acupuncture over conventional treatment. All reported high tolerability to acupuncture, including severely autistic children with behavioral symptoms. No specific significant side effects were reported in any of the studies reviewed. On the surface, it appears that acupuncture is effective and safe for treatment of symptoms of autism. However, a few critical caveats need to be considered when one evaluates these studies.

1. *Study Design.* The majority of the clinical studies were poorly designed and with low Jadad scores. Therefore, interpretation is limited.
2. *Assessment Bias. *All but four studies failed to report outcome assessment by blinded researchers. Of the four, only one was double blinded. It is known that acupuncture has high expectancy of receiving an effective treatment [57]. The reported superior efficacy of acupuncture over comparison treatment could be biased when the assessor knew the status of the patient's treatment. This bias became even a greater problem when the comparison was based on pre- and posttreatment.
3. *Control Groups.* The majority of the studies reviewed used behavioral intervention as a comparison group. While some of the subjects received only acupuncture and did not receive formal behavioral therapy during the treatment periods, it can be argued that some form of behavioral training was carried out consciously or subconsciously to the effect that the autistic children tolerated the entire course of acupuncture. Such training may have resulted in treatment effects that were not factored within the study results. In addition, two studies used sham acupuncture control groups. Sham acupuncture was performed by inserting an acupuncture needle into nonacupoints. This sham needling may induce local physiological and biochemical changes that could potentially lead to benefits similar to acupuncture [57]. Sham acupuncture had better efficacy than nontreatment arms.
4. *Randomization.* Many of the studies were not randomized. For example, some studies allowed parents to choose the modality of treatment (acupuncture versus others) [16–18, 24]. Major selection bias was inevitably introduced into the studies.
5. *Acupuncture Protocol.* There are no standard protocols in using an acupuncture treatment in autism. The choice of acupoints, the technique of acupuncture, and the length, frequency, and duration of treatment course were highly variable amongst the studies. The 17 studies that did not use Jin's three-needle system showed no consensus on the selection of acupoints. These variables are perceived to be important in acupuncture and make it difficult to compare studies.
6. *Outcome Measures.* The outcome measures used in the majority of the studies were largely subjective questionnaire checklists. Two studies used physiologic measurements that are presumably less subjective. One study used perfusion radiographic measurement [34], but it was not clear how the measurements obtained correlated with the acupuncture treatment. Another study [25] used electroencephalographic event-related potential but had only ten subjects.
7. *Age of Study Participants and Course of Treatment. *While many studies report greater improvement in younger children, the natural developmental progression in some of the young autistic children was not accounted for in some studies that last many months.

Autism spectrum disorders are a group of heterogeneous disorders, and many biomedical studies or treatment trials in autism spectrum disorders highlight subgroups with different responses to treatment. It is possible that a subgroup of autism spectrum disorders responds better to acupuncture. Yuan et al. [16, 19, 24, 25] attempted to identify such a subgroup by analyzing their results based on the severity of autism, the different TCM syndrome diagnosis of autism behaviors, the age of the subjects, and specific cognitive, behavioral, or developmental symptoms. They reported that age did not affect the results; the autistic children with higher CARS scores had greater improvement than those with lower CARS scores; and finally, greater improvement in autistic children with TCM types of liver-qi stagnation, hyperactivity of heart-liver fire, and phlegm blocking heart orifice occurred. 

Are acupuncture methods important for a better result? Could the choice of acupoints, the use of electric stimulation, the duration of needling, the cycle, or the length of the treatment course have any impact on improvement rate? The studies reviewed failed to provide direct answers to these questions. Some acupuncturists believe the longer the duration of the needling, the better the effects (personal communications). 

Tolerability or safety of acupuncture was not the focus of any of the studies reviewed. While serious side effects are rare when acupuncture is performed by an experienced acupuncturist, potential serious side effects can occur. A case report of dura puncture in an infant whose skull sutures were not fused was presented at the recent aforementioned conference stated. 

## 5. Conclusion

Acupuncture is frequently used in China to treat a variety of disorders including those with neurological dysfunction. In the United States, there is increased recognition of acupuncture as a treatment modality. Our review of published research on acupuncture in children with autism spectrum disorders serves to further the understanding of the application of acupuncture treatments for children with autism spectrum disorders. All of the reviewed articles invariably reported improvement in children with autism who received a form of acupuncture. All the clinical trials reported superior effects of acupuncture over comparison treatment. All reported high tolerability to acupuncture, including severely autistic children with behavioral symptoms. However, the studies reviewed have inherent weaknesses that made it difficult to interpret efficacy and safety. These weaknesses include poor study design, assessment bias, nonrandomization, variable acupuncture protocol, subjective outcome measures, and variable course of treatment. Continued research is needed to validate efficacy and safety of acupuncture. A multicenter controlled double-blinded study of acupuncture treatments in children with autism spectrum disorders is recommended.

## Figures and Tables

**Table 1:** Summary of the 27 clinical studies.

Study by reference	Number of subjects	Controls	Randomization	Blinded*	Description of subject dropout	Jadad Scores
Wong et al. [43]	55	Yes	Yes	DB*	Yes	5
Zhang et al. [22]	30	Yes	Yes	No	Yes	3
Allam et al. [45]	20	Yes	Yes	SB*	No	3
Li et al. [41]	70	Yes	Yes	SB*	No	2
Wong and Sun [44]	50	Yes	Yes	SB*	No	2
Yan et al. [30]	40	Yes	Yes	No	No	2
Zhang et al. [25]	30	Yes	Yes	No	Yes	2
Ma et al. [21]	44	Yes	Yes	No	No	1
Yuan et al. [16, 19, 24], Wu et al. [23]	202	Yes	No	No	Yes	1
Liu and Yuan [32]	67	Yes	Yes	No	No	1
Wang et al. [31]	60	Yes	Yes	No	No	1
Zhou and Zhang [37]	30	Yes	Yes	No	No	1
Li et al. [39]	38	Yes	Yes	No	No	1
Xie [26]	182	Yes	No	No	No	0
Yuan et al. [17]	69	Yes	No	No	No	0
Yuan et al. [20]	49	Yes	No	No	No	0
Yuan et al. [18]	40	Yes	No	No	No	0
Liu et al. [28]	38	No	No	No	No	0
H. Wu and Z. Y. [7], Wu [36]	35	No	No	No	No	0
Luo et al. [27]	35	No	No	No	No	0
Jia et al. [34]	34	No	No	No	No	0
Xi et al. [29]	32	No	No	No	No	0
Zhao et al. [42]	24	No	No	No	No	0
Ju and Feng [33]	13	No	No	No	No	0
Zhang [38]	12	No	No	No	No	0
Jiang and Wang [35]	11	No	No	No	No	0
Wang [40]	11	No	No	No	No	0

Total 27 studies	Ranges 11–202	17/27 studies had controls	12/27 studies randomized	4/27 studies were blinded	4/27 studies described dropouts	Jadad score 5→1 study 3→2 studies 2→4 studies 1→16 studies

*DS: double blinded, SB: single blinded.

**Table 2:** Method of acupuncture used.

Acupoints	Angle of needling	Depth of puncture	Duration of needling (minutes)	Frequency (weekly)	Length of treatment
Jin's three needles acupoints, body, or tongue acupoints	0°, 45°, 90°, or towards a specific anatomic site	0.1–1 *cun *	0, 0.4, 10, 25, 30, 45, 60, up to 240	Ranges from once to six times weekly	Ranges from 4 weeks to 9 months

**Table 3:** Prospective controlled clinical trials of acupuncture in autistic children with 50 or more subjects or Jadad score ≥2.

Study by reference^a^	Number of subjects	Age (yrs)	Length of study	Group design^b^	Jadad score	Outcome measures	Results
Wong et al. [43]	55	3–18	4 wks	EA versus SEA	5	WeeFIM, PEDI, Leiter-R, CGI-I, ABC, RFRLS, RDLS, parental report	Greater improvements of language, self-care and overall CGI-I scores, motor coordination, social skills, and attention span in EA group than these in SEA group (*P* < 0.006, *P* < 0.01)

Zhang et al. [22]	30	2–12	4 mos	ACP versus herbs	3	Chinese version of IQ, social, language tests	Greater improvement in all the measures after treatment in ACP group than that of herbs group (*P* < 0.05, *P* < 0.01)

Allam et al. [45]	20	4–7	9 mos	ACP + LT versus LT	3	Arabic language test	Both groups improved. Greater improvement in attention and receptive semantics in ACP + LT group (*P* = 0.008, 0.034, resp.)

Li et al. [41]	70	2–10	3 mos	ACP + BT + MT versus BT + MT	2	CARS, Clancy autism behavior scale, ABC, Gesell development scale	Significant improvement in all the measures before and after treatment in ACP + BT + MT group (*P* < 0.01). Better improvement in Gesell scale in ACP + BT + MT group (*P* < 0.05)

Wong and Sun [44]	50	3–11	8 wks	TAC versus sham	2	Griffiths mental developmental scale, WeeFIM, RFRLS, RDLS, symbolic play test	Improvements were documented in both groups, with the greater improvement in TCA group in some of the functional independence measures (*P* < 0.0005, *P* < 0.006)

Zhang et al. [25]	30	? –12	4 mos	ACP versus herbs	2	Event-evoked potential P3 latency and amplitude	Shortened latency and increased amplitude in ACP groups only after treatment (*P* < 0.001, *P* < 0.05). No change in herbs group

Yan et al. [30]	40	2.5–8	90 days	ACP + BT versus BT	2	C-PEP	Greater improvement in ACP + BT group than BT group (*P* < 0.01)

Liu and Yuan [32]	67	3–9	12 wks	ACP versus SIT	1	CARS and ABC	Greater improvement of both ABC and CARS in ACP group than that in SIT group (*P* < 0.05)

Wang et al. [31]	60	3–9	4 mos	EA versus BT	1	PPVT overall score and subscores	Greater improvements of overall score, subscores of sensation, association, body and self-care factors in EA group (86%) than those in BT group (56%) (*P* < 0.05)

Yuan et al. [16, 19, 24], Wu et al. [23]	202	1.5–8 yrs	4 mos	ACP versus BT	1	CARS (total and subscores)	88% of ACP group, 65% of BT group improved in CARS. ACP was effective across all age groups especially in more severe subjects, and in 3 of the 4 TCM syndromes. All CARS subscores except imitation and fine motor function were improved in both groups (*P* < 0.05, *P* < 0.01)

Xie [26]	182	<3 or >3	4 mos	ACP + TCM versus BT	0	Intelligence tests (DQ, WPPSI, WISC-R), clinical improvement scales (operationally defined)	Greater improvement of DQ, WPPSI, and clinical symptoms in ACP + TCM group; greater clinical improvement in <3 yrs old in both treatment groups compared with those of >3 yrs (*P* < 0.05, *P* < 0.01)

^a^The numbers in superscript are citation numbers corresponding to the numbers in reference section. ^b^All the subjects enrolled in any of the clinical trials were children with autism, both treatment and control groups. All studies in this table were prospective controlled trials.

Abbreviations in alphabetical order: ABC: aberrant behavioral checklist; ACP: acupuncture; BT: behavioral therapy; CARS: childhood autism rating scales; CGI-I: clinical global impression-improvement; C-PEP: Chinese version of psychoeducational profile; DQ: developmental quotient; EA: electroacupuncture; IQ: intelligent quotient; LT: language therapy; MT: music therapy; PEDI: pediatric evaluation development inventory; PPVT: Peabody Picture Vocabulary Test; RDLS: Reynell Developmental Language Scale; RFRLS: Ritvo-Freeman.

Real-life scale; SEA: sham electroacupuncture; SIT: sensory integration therapy; TAC: tongue acupuncture; TCM: traditional Chinese medicine; WeeFIM: functional independence measure for children; WISC-R: wechsler intelligence scale for children-revised; WPPSI: Wechsler preschool and primary scale of intelligence.
